# …..and the kidney biopsy solved the puzzle

**DOI:** 10.1007/s40620-022-01520-9

**Published:** 2022-12-15

**Authors:** Vittoria Esposito, Marco Colucci, Luca Semeraro, Ciro Esposito

**Affiliations:** 1 Unit of Nephrology and Dialysis, ICS Maugeri SpA SB, University of Pavia, Via Maugeri 10, 27100 Pavia, Italy; 2 Department of Internal Medicine and Medical Therapy, University of Pavia, Via Aselli 43/45, 27100 Pavia, Italy

**Keywords:** Glomerulonephritis, Kidney biopsy, Waldenstrom Macroglobulinemia, Pseudothrombi, Proteinuria, IgM

A 56-year-old male was referred because of edema and increased serum creatinine Two months earlier, he was admitted to the emergency room for dyspnea and edema and was diagnosed with severe aortic valve stenosis; urinalysis was unremarkable. He underwent aortic valve replacement with a mechanical valve and initiated anticoagulant therapy with warfarin. Afterwards, he was transferred to a cardiac rehabilitation ward and was later discharged in apparent good health. A few weeks after hospital discharge, because of elevated blood pressure values, calcium channel blockers were started and furosemide and spironolactone were further added because of lower limb edema. Laboratory tests showed increased serum creatinine (1.4 mg/dl). The nephrologist advised discontinuing calcium channel blockers and diuretics and starting therapy with doxazosin. However, three days later the patient was admitted to the Nephrology Unit because of the persistence of edema and the occurrence of macroscopic hematuria. On admission, physical examination confirmed moderate bilateral pitting edema, moderate hepatomegaly, and 3/6 heart murmur with no added sounds on thoracic auscultation. The remaining physical examination was normal. The laboratory tests showed: serum creatinine 1.68 mg/dl, Hb 9 g/dl, total serum protein 5.9 g/dl with normal serum electrophoresis, SGOT 58 U/l, SGPT 118 U/l, GGT 289 U/l, C3 50 mg/dl, C4 3 mg/dl, serum sodium 145 mmol/l, serum potassium 3.96 mEq/l, ERS 25 mm/h, CRP 0.1 mg/dl, normal immunoglobulin levels and proteinuria 1.4 g/l. Warfarin was discontinued and heparin therapy was started and a kidney biopsy was performed [1]. Light microscopy showed 16 hypercellular, lobulated glomeruli with intracapillary fuchsinophilic pseudothrombi (Fig. 1A–C). Immunofluorescence showed that the pseudothrombi were strongly positive for IgM and lambda chain. Fuchsin-positive deposits were found in the subendothelial space along the glomerular basement membrane (Fig. 1B–D). The diagnosis was membranoproliferative glomerulonephritis with intracapillary IgM deposits, most likely secondary to Waldenstrom macroglobulinemia (WM) [2]. The diagnosis was confirmed by serum immunofixation, which detected two small monoclonal components (IgM lambda 0.4 g/dl, and free lambda < 0.3 g/dl), in the presence of Bence Jones lambda type proteinuria and cryoglobulins mainly composed of IgM lambda free light chain. Cryocrit was 15%. A bone marrow biopsy was diagnostic for lymphoplasmacytic lymphoma. A computerized tomography scan showed para-aortic, retroperitoneal adenopathy. L265P mutation of the MY288 gene was found. Treatment with rituximab, cyclophosphamide, and dexamethasone led to complete remission of proteinuria and normalization of renal function. Waldenstrom macroglobulinemia is an uncommon B-cell lymphoproliferative disorder characterized by bone marrow infiltration and immunoglobulin M gammopathy. Common symptoms are anemia, hepatomegaly, splenomegaly, and lymphoadenomegaly, along with elevated B2 microglobulin levels. Kidney involvement is rare. Mild proteinuria and microscopic hematuria are common but unspecific renal manifestations of the disease. Kidney biopsy findings include AL amyloidosis, direct infiltration by lymphoid cells, and, as in our patient, cryoglobulinemic glomerulonephritis with intracapillary aggregates of IgM. Heart involvement is quite rare in Waldenstrom macroglobulinemia and is usually due to amyloid deposition, however, we cannot exclude that our patient’s heart valve disease could be related to this diagnosis.

**Fig. 1:**
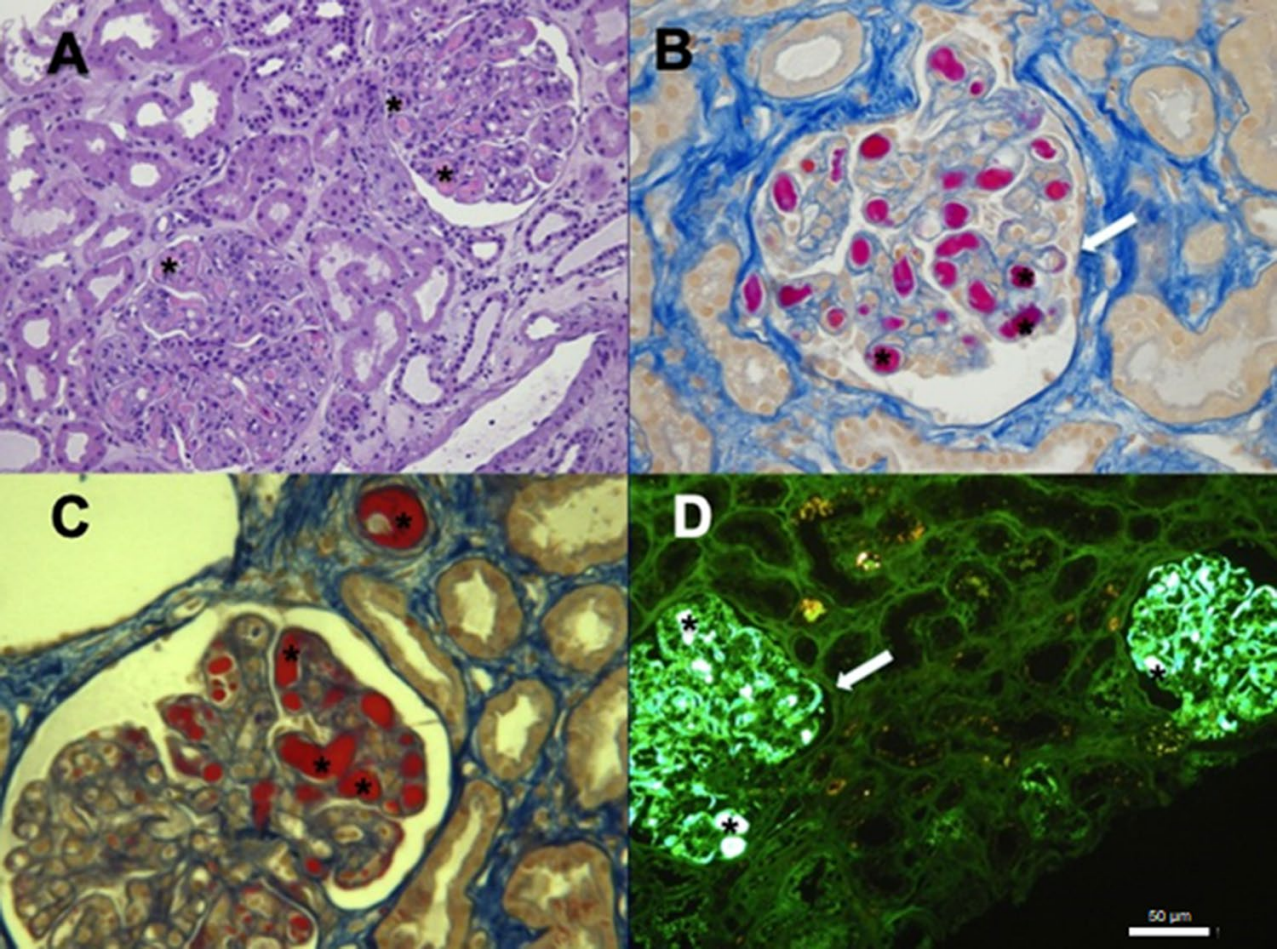
Light microscopy and immunofluorescence of the patient’s kidney biopsy sections. **A** Hematoxylin–Eosin (H&E) staining (100×) with several intracapillary pseudothrombi (*) and intracapillary proliferation; **B** Masson Trichrome and **C** Acid Fuchsin Orange G (AFOG) staining (400×) with many fuchsinophilic intracapillary pseudo-thrombi (*) which also occluded some small arteries (asterisks); **D** Immunofluorescence staining for IgM showing strong reaction in the intracapilllary pseudothrombi and deposits along the glomerular basement membranes (arrows)

## Data Availability

Data of the present case report is available at request and can be accessed from the patient’s chart.
